# Analysis of the Glycoside Hydrolase Family 1 from Wild Jujube Reveals Genes Involved in the Degradation of Jujuboside A

**DOI:** 10.3390/genes14061135

**Published:** 2023-05-24

**Authors:** Mingjun Yang, Yimian Ma, Xupeng Si, Xiaofeng Liu, Xin Geng, Xin Wen, Guoqiong Li, Liping Zhang, Chengmin Yang, Zheng Zhang

**Affiliations:** 1 School of Life Science and Engineering, Lanzhou University of Technology, Lanzhou 730050, China; yangmj@lut.cn (M.Y.); sixupeng0527@163.com (X.S.); 18009400938@163.com (X.L.); 2 National Engineering Laboratory for Breeding of Endangered Medicinal Materials, Institute of Medicinal Plant Development, Chinese Academy of Medical Sciences & Peking Union Medical College, Beijing 100193, China; ymma@implad.ac.cn (Y.M.); gx_0117@126.com (X.G.); lpzhang@implad.ac.cn (L.Z.); cmyang@implad.ac.cn (C.Y.)

**Keywords:** wild jujube, jujuboside, β-glucosidase, prokaryotic expression, molecular docking

## Abstract

Jujubosides are the major medicinal ingredients of Ziziphi Spinosae Semen (the seed of wild jujube). To date, a complete understanding of jujuboside’s metabolic pathways has not been attained. This study has systematically identified 35 β-glucosidase genes belonging to the glycoside hydrolase family 1 (GH1) using bioinformatic methods based on the wild jujube genome. The conserved domains and motifs of the 35 putative β-glucosidases, along with the genome locations and exon–intron structures of 35 β-glucosidase genes were revealed. The potential functions of the putative proteins encoded by the 35 β-glucosidase genes are suggested based on their phylogenetic relationships with *Arabidopsis* homologs. Two wild jujube β-glucosidase genes were heterologously expressed in *Escherichia coli*, and the recombinant proteins were able to convert jujuboside A (JuA) into jujuboside B (JuB). Since it has been previously reported that JuA catabolites, including JuB and other rare jujubosides, may play crucial roles in the jujuboside’s pharmacological activity, it is suggested that these two proteins can be used to enhance the utilization potential of jujubosides. This study provides new insight into the metabolism of jujubosides in wild jujube. Furthermore, the characterization of β-glucosidase genes is expected to facilitate investigations involving the cultivation and breeding of wild jujube.

## 1. Introduction

Glycosidases are hydrolases that are ubiquitous in organisms and do not require any coenzymes and cofactors to act on specific glycosidic bonds [1]. β-glucosidase (EC 3.2.1.21) (also known as β-D-glucosidase) belonging to glycoside hydrolase family 1 (GH1) catalyzes the hydrolysis of β-glycosidic bonds at the non-reducing end of the substrate to release D-glucose, which is involved in glycolipid metabolism in plants. D-glucose also contributes to cell wall degradation and activation of bioactive substances that play an essential role in maintaining the normal physiological functions of plants [2,3]. In plants, GH1 β-glucosidase acts on β-glucosides of various secondary metabolites, including plant carbohydrates, such as salicylic acid [4], tuberonic acid [5], abscisic acid [6], gibberellin [7], benzoxazinoids [8], cyanohydrins [9], alkaloids [10,11], and phenylpropanoids [12,13,14]. In recent years, an increasing number of β-glucosidase family members in various plant species have been reported, and the function of β-glucosidase genes has become more clearly understood. For example, 40 and 34 β-glucosidase genes belonging to the GH1 family have been discovered in *Arabidopsis* and *Oryza sativa*, respectively [15,16]. AtBGLU21-23 is related to the production of scopolamine in *Arabidopsis* roots [12,17]. AtBGLU18 and AtBGLU33 regulate ABA response by increasing ABA levels through the hydrolysis of glucose-binding ABA [6,18]. AtBGLU9 and AtBGLU10 catalyze the last step of anthocyanin formation in *Arabidopsis* [19]. AtBGLU42 is an MYB72-dependent key regulator of systemic resistance induced by rhizobacteria. It is mainly involved in systemic resistance induced by rhizobacteria through mild co-glycosylation of phenolic compounds (primarily coumarin β-glucoside and scopolamine) and regulates iron sensitivity in *Arabidopsis* roots [20,21]. Additionally, AtBGLU42 has a high sequence identity (64%) with rice cytoplasmic GH1 β-glucosidase Os1BGlu4. Os1BGlu4 has high hydrolysis activity for β-1,3-linked and β-1,4-linked cello-oligosaccharides for a degree of polymerization 3-4 in addition to β-glucosides, such as salicin and esculin. This indicates that AtBGlU42 can act on scopolamine and may also act on various β-glucosides, including oligosaccharides [22].

Wild jujube, or Chinese sour jujube [*Ziziphus jujuba* Mill. var. *spinosa* (Bunge) Hu ex H. F. Chow], is a representative subspecies of jujube (*Ziziphus jujuba* Mill.) in the Rhamnaceae family. Wild jujube is one of the oldest wild fruit trees or shrubs native to China. It is suitable for growing on hillsides and sandy saline–alkali land. It is a good variety for greening barren hills, improving sandy saline–alkali land, soil, and water conservation [23,24,25]. In some areas of Shanxi and Hebei provinces in China, wild jujube has become one of the most important economic crops [26,27]. The wild jujube fruit has elevated levels of vitamin C, is nutritionally rich, and can be used as a raw material for drinks or jam. Its fruit also has medical value and health-promoting functions, making it a new generation of wild fruit resources with excellent development potential [28]. Aside from its fruit, the seed of wild jujube (Ziziphi Spinosae Semen) is a well-known Chinese medicinal component [29,30]. Nowadays, people face increasing pressure, with the accelerated pace of modern life and the accelerated aging process. Insomnia, anxiety, and other mental health problems are commonly experienced by most individuals [31,32]. Ziziphi Spinosae Semen is a traditional Chinese therapeutic used to improve sleep quality and relieve nervousness [33,34]. For Ziziphi Spinosae Semen, jujuboside A (JuA) is one of the most important medicinal active ingredients. However, it has been suggested that JuA catabolites, including jujuboside B (JuB) and other rare jujubosides (especially jujubogenin), play more important roles in its medicinal efficacy [35]. Previous research has generally focused on jujuboside’s therapeutic properties, with limited investigations on jujuboside’s biosynthesis and catabolic processes. Since the public release of jujube’s (*Z. jujuba* Mill.) and wild jujube’s (*Z. jujuba* var. *spinosae*) genome, functional genomics research on jujube has been accelerated [36,37]. Some studies have focused on genes involved in the biosynthesis, accumulation, or regulation pathways of the jujube triterpenoids and volatiles [28,38,39,40,41]. However, the key enzyme genes involved in the catabolic pathway of jujubosides in wild jujube remain unknown. Based on publicly available genomic data, this study reveals 35 β-glucosidase genes of wild jujube and analyses their gene structures and chromosome locations. The functions of the putative proteins encoded by the *ZsBgl* genes are predicated by their classified groups, based on the phylogenetic analysis of the *Arabidopsis* GH1 family β-glucosidases. Four *ZsBgl* genes are cloned and heterologously expressed in *E. coli*. Following a short IPTG induction, two of the recombinant *Escherichia coli* stains carrying *ZsBgl03* and *ZsBgl24* can produce the expected proteins able to catalyze the deglycosylation of JuA. This study identifies GH1 family β-glucosidase genes in wild jujube, and experimentally validates the efficacies of two *ZsBgl* genes for JuA catabolism. The enhanced understanding of the secondary metabolic pathways of wild jujube may favorably improve the economic value of wild jujube and Ziziphi Spinosae Semen.

## 2. Materials and Methods

### 2.1. Plant Materials

Wild jujube seeds were purchased from the market and have been identified by Professor Zheng Zhang as *Z. jujuba* Mill. var. *spinosa* (Bunge) Hu ex H. F. Chow (wild jujube or Chinese sour jujube). These seeds were planted in sterilized soil and cultivated in a greenhouse (ambient light; temperature 25 °C; relative humidity 70%) for approximately 1 month, until they reached about 10 cm in height. Seedlings were then used as materials for RNA extraction.

### 2.2. Acquisition of the Sequences of Potential β-Glucosidases of Wild Jujube

The files of the coding sequences and putative proteins of wild jujube were downloaded from the databases of the National Center for Biotechnology Information with the BioProject accession number of PRJNA840890. The Hidden Markov model (HMM) profiles of the BH1 (PF00232) were extracted from the Pfam database (http://Pfam.sanger.ac.uk, accessed on 11 May 2022) to find potential β-glucosidases of wild jujube. Searches with the HMM model were conducted, using the hmmsearch program from the HMMER package, against putative proteins of wild jujube. The significant hits (E-value < 10^−10^) were identified as candidate β-glucosidases of wild jujube. All candidate sequences were then submitted for domain analysis using the SMART tool (http://smart.embl-heidelberg.de/, accessed on 11 May 2022) and the web CD-Search Tool (https://www.ncbi.nlm.nih.gov/Structure/bwrpsb/bwrpsb.cgi, accessed on 11 May 2022) in the National Centre for Biotechnology Information (NCBI) for further validation. Thirty-five proteins remained after redundant and repetitive sequences were deleted.

### 2.3. Phylogenetic Relationship Analysis

A phylogenetic tree for 82 β-glucosidases from wild jujube and *Arabidopsis* was constructed using the neighbor-joining (NJ) method implemented in MEGA 11 [42]. The MUSCLE program performed the multiple sequence alignment of the 82 β-glucosidase sequences. The parameters for tree construction used bootstrap values of 1000 replicates and default parameters. Finally, the phylogenetic tree was modified using the online Evolview tool (http://www.evolgenius.info/evolview/#/treeview, accessed on 6 March 2023) [43].

### 2.4. Chromosome Location and Gene Structure Analysis

Locations of genes encoding putative β-glucosidases of *Z. jujube* var. *spinosa* (named *ZsBgl* genes) were obtained from the genome annotation information of *Z. jujube* var. *spinosa* (Genbank, GCA_020796205.1). The locations of *ZsBgl* genes were illustrated using the TBtools. The numbers and organization of introns, exons, and gene structures were drawn and displayed using the TBtools software [44]. The online MEME suit 5.5.2 (https://meme-suite.org/meme/tools/meme, accessed on 7 March 2023) was subsequently used to investigate the putative conserved motifs among ZsBgl proteins, using default parameters.

### 2.5. Analysis of Physicochemical Properties Concerning the Putative ZsBgl Proteins

The subcellular localization of ZsBgl proteins was predicted using WoLF PSORT II (http://psort.hgc.jp/form2.html, accessed on 1 March 2023). The pI/MW calculation tool of ExPASy (http://web.expasy.org/compute_pi/, accessed on 1 March 2023) was used to predict pI and molecular weight.

### 2.6. RNA Isolation and Reverse Transcription PCR

Wild jujube’s fresh and robust seedlings (with their roots cut) were selected to isolate total RNA. The total RNA of the wild jujube was extracted using the RNAprep Pure Plant Plus Kit (Tiangen, Beijing, China). The RNA integration was analyzed using a 1% agarose gel, and the total RNA was quantified using a NanoDrop2000 spectrophotometer (Thermo Scientific, Waltham, MA, USA). Reverse transcription was undertaken using the TIANScript II RT Kit following the manufacturer’s instructions (Tiangen, Beijing, China).

### 2.7. Heterologous Expression of Two ZsBgl Genes in E. coli

The prokaryotic expression vector was constructed using the EasyGeno Assembly Cloning kit (Tiangen, Beijing, China). The coding sequences of *ZsBgl03* and *ZsBgl40* were amplified by reverse transcription PCR (RT-PCR) with gene-specific primers, shown in Table S1. Two sequences were recombined into the pET32a vector using the recombination primers shown in Table S2. The RT-PCR program used was 1 cycle of 94 °C for 3 min; 35 cycles of 94 °C for 30 s, 53 °C for 30 s, and 72 °C for 3 min, followed by a final extension of 72 °C for 10 min in a thermal cycler (Thermo Scientific, Waltham, MA, USA). For gene recombination and seamless ligation, the pET32a vector (Novagen, Reno, NV, USA) was linearized using restriction enzymes Xho I and Nco I and recombined with the coding sequence of *ZsBgl03* and *ZsBgl40*. The constructed plasmid was transformed into *E. coli* Rosetta-gami (DE3) plysS cells. The recombined *E. coli* was induced using 0.2 mM isopropyl β-D-thiogalactoside (IPTG) for 1 h, 2 h, 3 h, and 4 h at 28 °C. The *E. coli* was then collected by centrifugation and treated with ultrasonic crushing. The bacterial lysates were checked using a sodium dodecyl sulphate-polyacrylamide gel electrophoresis (SDS-PAGE), and a protein band of the expected molecular weight in the expressed proteins of the bacteria confirmed the fusion protein expression. The experimental process is similar to that described by Zhang et al. [45].

### 2.8. Determination of Enzymatic Activity Using HPLC

After IPTG induced the recombined *E. coli* for 4 h, 20 mL of the bacterial solution was centrifuged at 4000 rpm, and the precipitate was resuspended and sonicated in 2 mL PBS buffer (10 mM Na_2_HPO_4_, 2 mM NaH_2_PO_4_, 135 mM NaCl, 4.7 mM KCl, pH 7.3). Then, 1.5 mL of the supernatant was centrifuged at 12,000 rpm, and 0.5 mL of 0.1 mg/mL JuA standard solution was added. The mixture was incubated at 28 °C for 2 h, and 1 mL incubation solution was then taken, filtered through a 0.22 μm filter membrane, and loaded into a 2 mL liquid injection vial for Shimadzu LC-2030 High-performance liquid chromatography (HPLC) determination, using a ZORBAX Eclipse XDB-C_18_ (4.6 × 250 mm, 5 μm) chromatographic column. The injection volume was 20 μL, the mobile phase was acetonitrile-water (34:66), and the detection wavelength was 203 nm. The column flow rate was 1 mL/min, and the column temperature was 30 °C.

### 2.9. UPLC-Oribtrap-Exploris-120-MS/MS Analyses

UPLC-Oribtrap-Exploris-120-MS/MS analyses were performed using a UHPLC system (Vanquish, Thermo Fisher Scientific, Waltham, MA, USA) with a UPLC ACQUITY UPLC BHE C18 column (2.1 mm × 100 mm, 1.7 μm) coupled to an Orbitrap Exploris 120 mass spectrometer (Orbitrap MS, Thermo, Waltham, MA, USA). The mobile phase consisted of acetonitrile and 0.1% aqueous formic acid (34:66), with a 0.2 mL/min flow rate. The injection volume was 1 µL, and the column temperature was 30 °C. The Orbitrap Exploris 120 mass spectrometer was used for its ability to acquire MS/MS spectra using the acquisition software (Xcalibur, Thermo, Waltham, MA, USA). In this mode, the acquisition software continuously evaluates the full-scan MS spectrum. The ESI source conditions were set as follows: The ion source was the ESI source, and positive and negative ions were scanned separately. The capillary voltages were 3.2 kV (ESI+) and 2.5 kV (ESI−), the ion source temperature was 320 °C, the auxiliary heater temperature was 350 °C, the sheath gas flow rate was 35 Arb, the Aux gas flow rate was 10 Arb, the capillary temperature was 320 °C, the full MS resolution was 120,000, and the MS/MS resolution was 15,000. The collision energy was 30%, 40%, and 50% in NCE negative ion mode and 50% in NCE positive ion mode.

### 2.10. Bioinformatic Analysis of the Putative Proteins and Molecular Docking

Molecular docking predicts the specific binding form of proteins with small molecule ligands. The Swiss-Model server (https://swissmodel.expasy.org, accessed on 19 March 2023) initially predicted the protein structure using homology modeling and selecting templates with coverage greater than 90%. The possible protein active catalytic pocket was then predicted using POCASA 1.1 (https://g6altair.sci.hokudai.ac.jp/g6/service/nocasa/, accessed on 19 March 2023), through visual analysis of the pocket data, combined with Pymol (http://www.pymol.org/, accessed on 19 March 2023). Finally, molecular docking was performed according to the binding space and the pocket with the highest retention possibility within the catalytic center. Discovery Studio 2019 Client software was used to predict the docking possibilities of β-glucose with the predicted protein pocket structure. The prediction range was the position of the pocket. Finally, Pymol was used to illustrate the details of the docking structure.

## 3. Results

### 3.1. Characteristics of ZsBgl Genes and Their Encoded Proteins

**Figure 1:**
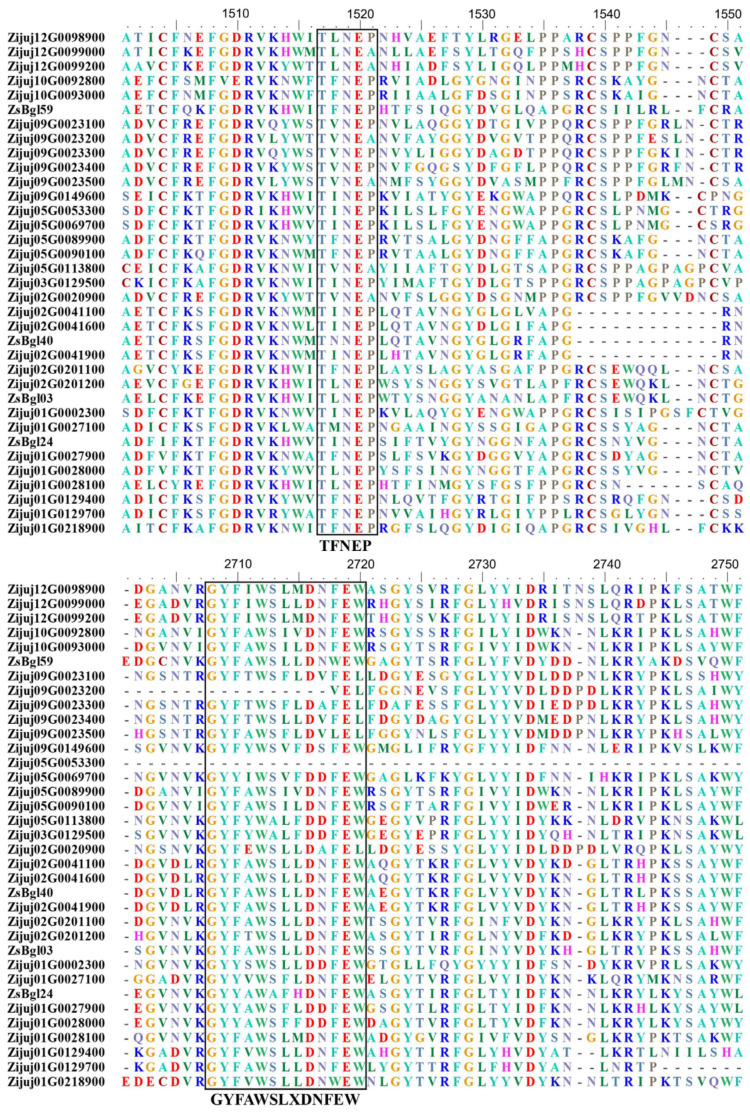
Schematic representation of the conserved motifs of putative ZsBgl proteins, extracted from the sequence alignment file of ZsBgl proteins.

So far, the β-glycosidases of wild jujube remain largely unidentified. This study used the hmmsearch program from the HMMER package to investigate the putative β-glycosidases from the putative proteins of wild jujube downloaded from Genbank (as described in the materials and methods). Finally, 35 putative β-glycosidases of wild jujube were identified. Through sequence alignment, it was revealed that almost all β-glucosidases contain the same conserved motifs of the Pfam domain (PF00232, GH1), including TFNEP (acid/base catalyst), IVTENG, and GYIFWTISDNWEW (an almost unchanged variant of GYFAWSLXDNFEW). These putative wild jujube β-glycosidases were named ZsBgl proteins, and their conserved protein motifs are shown in the truncated sequence alignment file (Figure 1). The genes encoding the 35 putative β-glycosidases of wild jujube were named according to their original IDs in the sequence file. The four cloned β-glycosidase genes from the wild jujube seedlings were named *ZsBgl59*, *ZsBgl40*, *ZsBgl03*, and *ZsBgl24*. From protein sequence analysis data, all predicated ZsBgl proteins contain the conserved glycosyl hydrolase 1 superfamily domain (GenBank accession cl14647), and some include additional domains. For example, protein Zijuj12G0099200 contains an additional BglB domain at its C-terminal (Figure S1). All genes have been searched against the Genbank nucleotide database, and accession numbers have been acquired. The Genbank accession numbers and the nucleotide sequences of the *ZsBgl* genes, as well as physicochemical properties of their encoded proteins, are shown in Table S3, along with their cellular location information. Overall, the length range of the *ZsBgl* gene coding sequence is 1005~3849 bp, and the ZsBgl proteins’ length range is 334~1282 amino acids. The ZsBgl proteins are primarily acidic, and the molecular weight is 37.63~144.07 kDa. The instability indexes for these proteins were less than 40, indicating that they are relatively stable. The grand averages of hydropathicity (GRAVY) for the proteins were negative, inferring they are all hydrophilic proteins. The proteins are predicted to localize in various cellular positions, with 10 localized in the cytoplasm and 15 in the chloroplast. Others are localized in plasma, mitochondria, and vacuolar. The predicated location of these proteins reflects their diversified functions in wild jujube, as has been reported for other species [46,47].

### 3.2. The Locations and Structures of ZsBgl Genes and Conserved Motif Information for ZsBgl Proteins

Wild jujube has a total of 12 chromosomes. The 35 *ZsBgl* genes are located on 7 chromosomes, but not on chromosomes 4, 6, 7, 8, and 11 (Figure S2). There are 9 *ZsBgl* genes on chromosome 1, the largest on a single chromosome, with only 1 gene on chromosome 3. *ZsBgl03* and *ZsBgl40* are located on chromosome 2. The exon–intron structural analysis shows that all the *ZsBgl* genes have multiple introns, with most having more than ten introns (Figure S3). Notably, the β-glycosidase genes of *Arabidopsis* also have multiple exons. They exhibited 10 distinct exon–intron organization patterns, with the 13 exon patterns being the most common [16]. The intron sizes and numbers of the *ZsBgl* genes are highly variable, consistent with reports for *Arabidopsis* and rice [15,16]. The conserved motifs of the predicted ZsBgl proteins were discovered using the online MEME tools, with ten conserved motifs predicted for each protein. All ZsBgl proteins contain the conserved motif 1 (Figure S4). Sequence analysis identified motif 1 as including the conserved core sequence “TFNEP”, which is also the core sequence of the conserved glycosyl hydrolase 1 superfamily domain (PF00232), as shown in the alignment file of Figure 1. The other core sequence of the conserved GH1 family domain (PF00232) identified in the alignment file of Figure 1, “GYFAWSLXDNFEW”, appears in the conserved motif 2. The motif analysis also shows that some conserved motifs are absent in specific ZsBgl protein sequences. For example, Zijuj09G0023200 and Zijuj05G0053300 do not contain motif 2, Zijuj09G0023200 does not have motif 5, and Zijuj03G0129500 and Zijuj02G0201200 genes do not contain motif 4. Motif 7 only exists in some ZsBgls, indicating these ZsBgls may share some relationships in systemic evolution.

### 3.3. Phylogenetic Relationships of β-Glucosidases from Wild Jujube and Arabidopsis

**Figure 2:**
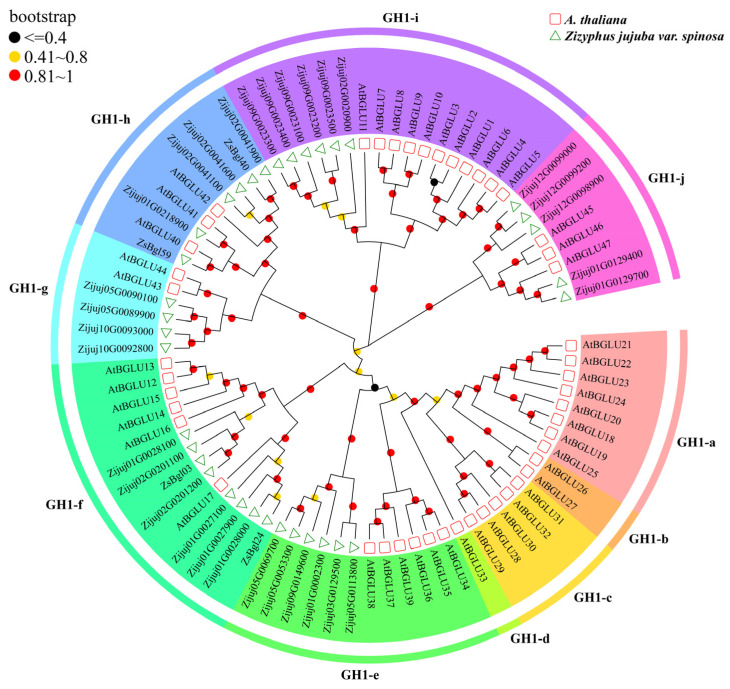
The neighbour-joining (NJ) tree shows the ten β-glycosidases groups identified from wild jujube and *Arabidopsis*.

A phylogenetic tree was constructed to elucidate the relationships of the ZsBgl proteins with β-glycosidases from *Arabidopsis* (AtBgl proteins). The AtBgl protein sequences were downloaded from the TAIR database (https://www.arabidopsis.org/, accessed on 25 February 2023), and the phylogenetic tree of 35 ZsBgl and 47 AtBgl proteins was constructed using the neighbour joining (NJ) method. As shown in Figure 2, 82 plant β-glucosidases are classified into 10 groups, from GH1-a to j, using the classification of plant β-glucosidases defined by Xu et al., where the plant β-glucosidases in the same group may have similar functions [16]. Thirty-five ZsBgl proteins are classified in this study across six phylogenetic groups, inferring that these proteins have similar functions to the AtBGLU proteins in the respective groups. For example, six ZsBgl proteins belong to the GH1-e class and may be involved in defense against herbivores, pathogen attacks, and abiotic stresses, such as that reported for AtBGLUs [48,49,50]. Eight ZsBgl proteins in the GH1-f group may be related to using flavonoids [14]. Four ZsBgl proteins belonged to GH1-g, which may also have mannosidase activity [51]. Six ZsBgl proteins belonged to GH1-h, which may induce systemic resistance to bacterial disease and pollen development [21,52]. Six ZsBgl proteins were grouped into the GH1-i branch, which may be involved in flavonol accumulation and anthocyanin biosynthesis [14,19,53]. Five ZsBgl proteins were grouped into the GH1-j branch, which may regulate lignin biosynthesis [54,55].

### 3.4. The Prokaryotic Expression of Two ZsBgl Genes

The four β-glycosidase genes cloned from wild jujube seedlings, *ZsBgl59*, *ZsBgl40*, *ZsBgl03*, and *ZsBgl24*, were recombined into the pET32a vector and transformed into *E. coli*. Only *ZsBgl03* (Genbank accession number XM_016020485.3) and *ZsBgl40* (Genbank accession number XM_048468469.1) were expressed as soluble proteins in *E. coli*, while the other two were expressed as inclusion bodies in cells. SDS-PAGE results showed that after 1 h of IPTG induction, the recombined ZsBgl03 and ZsBgl40 proteins were expressed in the supernatant, and the recombined ZsBgl proteins reached maximum quantities after 3~4 h of IPTG induction (Figures S4 and S5). The expression of the two ZsBgl proteins was estimated by comparison to the molecular weight marker concentration. The results showed that following induction by 0.2 mM IPTG for 4 h, the concentration of ZsBgl03 and ZsBgl40 fusion proteins were approximately 0.2 mg/mL and 0.3 mg/mL, respectively. Through sequence analysis, the coding sequence for *ZsBgl03* was 1485 bp (encoding 494 amino acids), while for *ZsBgl40*, the coding sequence was 1497 bp (encoding 498 amino acids). The phylogenetic analysis data predicted that ZsBgl03 may belong to the GH1-f group and participate in flavonoid metabolism [14,56]. ZsBgl40 may belong to the GH1-h group and be involved in defense mechanisms and systemic resistance to bacterial disease [52,57]. Using the WoLF PSORT II software, it was predicated that ZsBgl03 mainly localized in the chloroplast, cytoplasm, endoplasmic reticulum, and nucleus, while ZsBgl40 localized in the nucleus and cytoplasm. Homologous protein searches against proteins from the NCBI non-redundant protein database (Nr database) using the BLASTp program showed that ZsBgl03 had 69.98% identity with the BT93_B3085 protein (GenBank accession number KAF8041056.1) from *Corymbia citriodora* subsp. *Variegate*, and ZsBgl40 identified 84.76% with glycoside hydrolase from *Quercus lobata* (XP_030928088.1). No previous studies have described the function of ZsBgl03 and ZsBgl40, including their homologues. Since these two genes were predicated to be involved in secondary metabolic and defense reactions, their functions in the degradation of JuA was tested.

### 3.5. Determination of the Activity of ZsBgl03 and ZsBgl40

**Figure 3:**
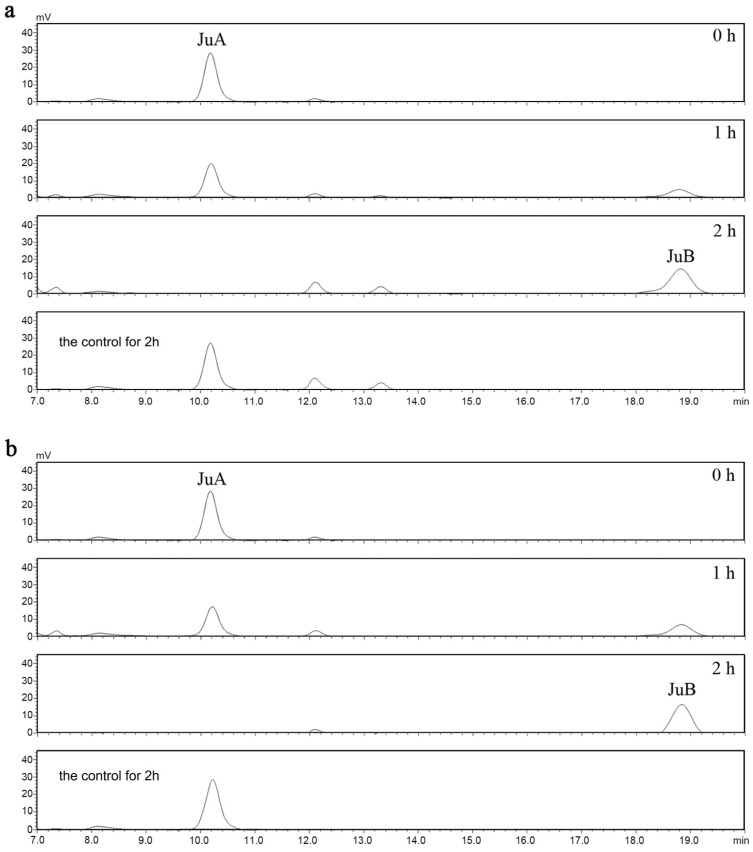
The HPLC chromatograms for the recombined *E. coli* carrying *ZsBgl03* and *ZsBgl40*. (**a**) HPLC chromatogram analysis of the JuA conversion by the recombinant *E. coli* carrying *ZsBgl03*, and (**b**) HPLC chromatogram analysis of the JuA conversion by the recombinant *E. coli* carrying *ZsBgl40*.

**Figure 4:**
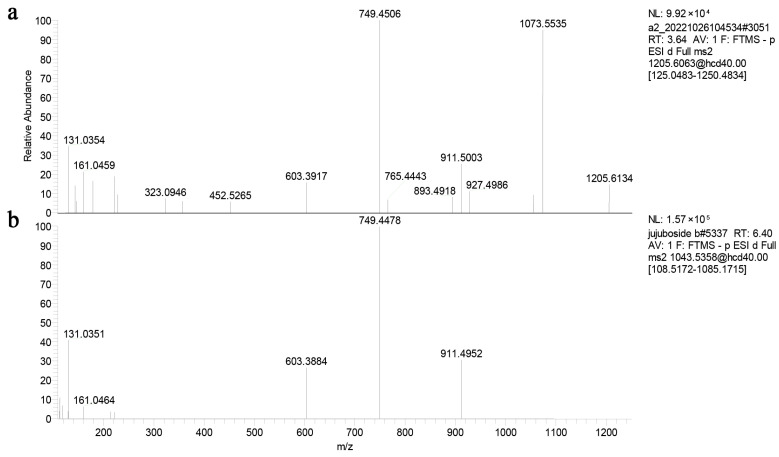
The fragmentation pathways of JuA and JuB residue ions in the negative MS/MS. (**a**) The fragmentation pathways of JuA residue ion in the negative MS/MS, and (**b**) the fragmentation pathways of JuB residue ion in the negative MS/MS.

In order to evaluate the deglycosylation capabilities of recombined ZsBgl03 and ZsBgl40, an analysis was conducted to determine the efficacy of these proteins to degrade JuA. Briefly, after IPTG induction for 4 h, cells in the supernatants of recombined and control *E. coli* were collected and incubated with 0.5 mL of 0.1 mg/mL JuA standard solution in PBS. After incubation for 1 h and 2 h, the reaction was stopped, and the reaction products were characterized using UPLC-MS/MS. The results showed that recombinant ZsBgl03 and ZsBgl40 enzymes generated a major product after two hours of incubation with JuA, which is identified as JuB by comparison to its retention times and mass spectra with authentic standards (Figure 3 and Figure 4). The conversion rates of ZsBgl03 and ZsBgl40 enzymes after 2 h of incubation with JuA were calculated to be approximately 86.9% and 78.8%, respectively.

Previous studies have identified saponins in the extract of Ziziphi Spinosae Semen [58,59,60]. In this study, we identified 15 saponins in the Semen Ziziphi Spinosae extract using the UPLC-MS/MS method (Table S4), in accordance with previous studies. Based on the saponins discovered, the transformation of JuA to jujubogenin requires five steps and five different enzymes, allowing for the biotransformation pathways of jujubosides to be deduced and shown as Figure S6.

### 3.6. Protein Structure Prediction and Molecular Modeling

**Figure 5:**
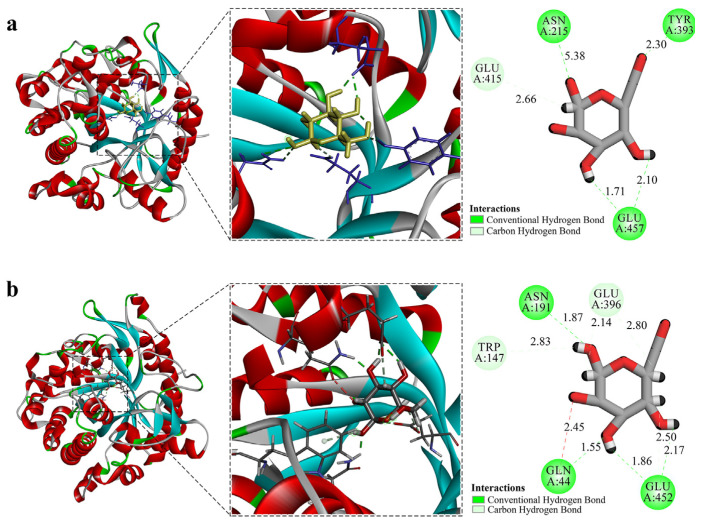
Predicted tertiary structures of ZsBgl03 and ZsBgl40 and their molecular docking models. (**a**) Predicted tertiary structure of ZsBgl03 and docked ligand; (**b**) Predicted tertiary structure of ZsBgl40 and docked ligand.

The online tool POASA 1.1 was used to predict the functional pockets of ZsBgl03 and ZsBgl40 (Figure S7). The active pocket with the most extensive volume has the highest likelihood of being the ligand-binding pocket. Visual analysis and molecular docking are used to rule out the possibility of false pockets and are highly conserved. Ligand docking was analyzed for ZsBgl03 and ZsBgl40 to explore and visualize the binding of substrates to the modeled active site. The ZsBgl03 protein has 167 amino acids in the α-helix secondary structure and 35 in the β-turn. There are 183 amino acids in the α-helix and 41 in the β-turn of the ZsBgl40 protein. Although they share only 51.84% sequence identity, both enzymes exhibited the same (β/α)8-barrel fold, with the active site at the barrel’s C terminus [61]. Hydrolysis of the β-glycosidic bond usually involves the participation of two catalytically active glutamate residues embedded in a highly conserved TFNEP (acid/base catalyst) and I/VTENG (nucleophile) peptide motifs [62]. These two proteins were docked with β-D-glucose to predict whether ZsBgl03 and ZsBgl40 proteins have a wide range of catalytic activity (Figure 5). The results showed that ZsBgl03 had a larger active pocket, which is tight and hydrophobic. Visual verification confirmed that it was not a false pocket, and the relatively tight internal space was larger than the pocket. Numerous H-bonds with the sugar-OH groups induce the active site specificity for β-glucosides. ASN215, TYR393, and GLU457 form H-bonds at positions within the active site. There are hydrogen bonds between glucose and the GLN 44, ASN191, GLU396, and GLU452 residues in the catalytic pocket of ZsBgl40. The docking and molecular structures revealed the possibility of the two proteins catalyzing other compounds, and β-D-glucose, at the molecular level.

## 4. Discussion

β-Glucosidase has specific substrate hydrolysis characteristics and essential application prospects in functional oligosaccharides preparation, fruit and vegetable preservation, biomedicine, and plant disease resistance [3,63,64]. Previous reports have investigated the structure, function, and applications of different β-glucosidase families. In this study, β-glucosidases in the GH1 family of wild jujube were analyzed for the first time, and 35 β-glucosidase genes were identified. According to the phylogenetic relationship between β-glucosidases in *Arabidopsis* and wild jujube, the putative ZsBgl proteins (β-glucosidases of wild jujube) were divided into six groups. The putative ZsBgl proteins may be related to plant hormone activation, pollen development, secondary metabolism, and plant defense against biotic and abiotic stresses. Such functionality is essential for studies on the growth and development of wild jujube. Recently, functional genomic studies on wild jujube have been accelerated. Through protein–protein interaction predictions and homology comparisons, it has been recognized that bHLH family genes in wild jujube have crucial functions during flower development [65]. The bioinformatic analyses of the bZIP family of wild jujube were also performed systematically. Their expression profiles showed that many genes might play crucial roles during fruit ripening and in response to phytoplasma abiotic stresses [66]. These studies may contribute to improving our understanding of the growth and metabolism of wild jujube. Identification of β-glucosidase genes in this investigation will provide significant target gene resources to change metabolic pathways and metabolite contents. The advancements in this field may help improve the cultivation and breeding techniques for wild jujube.

Phylogenetic analysis inferred that ZsBgl03 belongs to the GH1-f group, and ZsBgl40 belongs to the GH1-h group. According to the classification of possible functions of each group, ZsBgl03 may be involved with flavonoid metabolism, and ZsBgl40 correlated with bacterial disease resistance. As members of the β-glucosidase family, they may all be capable of hydrolyzing glycosidic linkages, but their substrates and transformation efficiencies remain unknown. JuA, which has a glucose group at the end of the sugar chain, is a very important natural sedative, hypnotic, and antitumor drug in wild jujube [33]. It has been previously reported that the catabolites of JuA, such as JuB and other rare jujubosides, may have further important medicinal value [35]. The heterologous expression of two ZsBgl proteins in *E. coli* first provided in vivo evidence of their efficacy in transforming JuA into JuB. According to our analysis, the conversion rate of ZsBgl03 was about 86.9%, and ZsBgl40 was approximately 78.8%. Considering the proteins’ predicted three-dimensional molecular structures, the catalytic pocket volume of ZsBgl03 was larger than ZsBgl40, as shown in Figure S6. Therefore, small molecule ligands can bind to ZsBgl03 more easily. This may be the reason why the catalytic activity of ZsBgl03 is relatively higher than ZsBgl40. To date, 55 triterpenoid saponins from wild jujube have been isolated and identified, predominantly tetracyclic and pentacyclic triterpenoids [28]. All jujubosides are naturally present in wild jujube. However, apart from JuA, all other contents were minimal. Rare saponins have crucial medicinal value [58]; however, it is unrealistic to obtain many other rare jujubosides by extraction from wild jujube fruits. Therefore, preparing other rare jujubosides using the *E. coli* strain constructed in this study would be more efficient and reliable. Recently, given that Ziziphi Spinosae Semen preparations can reduce nervousness and stress levels, the demand for these preparations has been increasing, both in China and around the world. However, there has been a gradual decline in the availability of Ziziphi Spinosae Semen, consequently leading to a significant annual increase in its price. This study may improve the utility value of jujubosides, providing products with higher added value to the wild jujube industry, and thereby contributing to clinical needs.

## 5. Conclusions

This bioinformatic analysis identified 35 *ZsBgl* genes in the GH1 family based on the genome of wild jujube and systematically analyzed their gene structure, physicochemical properties, phylogenetic relationships, conserved motifs, and chromosomal distributions, which has provided deep insights into this gene family. Notably, two *ZsBgl* genes named *ZsBgl03* and *ZsBgl40* were cloned and characterized. Their efficacy to transform JuA was identified using heterologous expression in *E. coli* and subsequent chemical analysis. These data confirm that the two genes can produce recombinant proteins capable of transforming JuA to JuB. The tertiary structures of ZsBgl03 and ZsBgl40 were predicted, and molecular docking models were proposed to explain their specific functions on a molecular level. The efficacy of the two enzymes in converting jujubosides illustrates the potential of jujubosides, enabling the development of byproducts with a higher economic value for the wild jujube industry. This study enhances the understanding of the functions of plant β-glucosidases in the GH1 family and has promising implications for the cultivation and breeding of wild jujube.

## Data Availability

The wild jujube resources were downloaded from the NCBI SRA database with accession number GCA_020796205.1. The protein sequences of *Arabidopsis* were downloaded from the TAIR database (https://www.arabidopsis.org/) according to their names or accession numbers. All data generated or analyzed during this study are included in this article and its supplementary materials.

## Supplementary Materials

The following supporting information can be downloaded at: https://www.mdpi.com/article/10.3390/genes14061135/s1. Table S1: *ZsBgl03* and *ZsBgl40* gene cloning primers; Table S2: *ZsBgl03* and *ZsBgl40* homologous recombination primers; Table S3: Characters of *ZsBgl* genes and their encoded proteins; Table S4: Mass data for the 15 saponins detected in the Ziziphi Spinosae Semen extract by UPLC-MS/MS; Figure S1: The major domains of the putative ZsBgl proteins; Figure S2: The exon/intron structures of the *ZsBgl* genes; Figure S3: The locations of the ZsBgl genes on the chromosomes; Figure S4: Detection of the heterologous expression of ZsBgl03 through SDS-PAGE; Figure S5: Detection of the heterologous expression of ZsBgl40 through SDS-PAGE; Figure S6: The biotransformation of jujubosides; Figure S7: The catalytic pocket and position in the tertiary structure of ZsBgl03 and ZsBgl40 predicted by the online tool POASA 1.1.
