# Supplementary material for: Analysis of the Glycoside Hydrolase Family 1 from Wild Jujube Reveals Genes Involved in the Degradation of Jujuboside A
Source: Genes (Basel). 2023 May 24;14(6):1135. doi: 10.3390/genes14061135 (PMC10298349; doi:10.3390/genes14061135)
Supplement: Supplementary file 1 [file genes-14-01135-s001.zip › genes-2381107-supplementary.pdf]

Table S1. *ZsBgl03* and *ZsBgl40* gene cloning primers

Primer	Sequence
ZsBgl03-F	ATGGCATCCCATTCGGGC
ZsBgl03-R	TTATCTTTTGAGGAAAGCCTTGAACC
ZsBgl40-F	ATGCCCAAGAAGGAGAACTTC
ZsBgl40-R	TTACTCGCCCTTGCCATTTTT

Table S2. *ZsBgl*03 and *ZsBgl*40 homologous recombination primers

Primer	Sequence
p-ZsBgl03-F	ACCGACGACGACGACAAGGCCATGGCATCCCATTCGGGC
p-ZsBgl03-R	AGTGGTGGTGGTGGTGGTGCTTATCTTTTGAGGAAAGCCTTGAACC
p-ZsBgl40-F	ACCGACGACGACGACAAGGCCATGCCCAAGAAGGAGAACTTC
p-ZsBgl40-R	AGTGGTGGTGGTGGTGGTGCTTACTCGCCCTTGCCATTTTT

Table S3 Characters of *ZsBgl* genes and their encoded proteins

Genbank accession number	Gene Name	CDS Length (bp)	Amino Acids (aa)	Molecular Weight (kDa)	PI	Instability index	Aliphaticin dex	Grand average of hydropathicity (GRAVY)	Cell location
XM_016043586.3	*Zijuj12G0098900*	1473	490	56.11	6.45	39.79	84.82	-0.229	Cytoplasm
XM_048465951.1	*Zijuj12G0099000*	3048	1015	116.48	5.53	35.50	79.57	-0.286	Cytoplasm
XM_048464783.1	*Zijuj12G0099200*	1596	531	61.01	6.71	40.95	85.01	-0.229	Chloroplast
XM_048462143.1	*Zijuj10G0092800*	1479	492	56.11	9.49	37.52	76.14	-0.321	Cytoplasm
XM_048462143.1	*Zijuj10G0093000*	1776	591	67.71	6.35	32.46	78.22	-0.282	Cytoplasm
XM_048462600.1	*ZsBgl59*	1455	484	55.14	8.34	26.88	81.03	-0.269	Vacuolar
XM_016036387.3	*Zijuj09G0023100*	1503	500	56.24	5.31	32.83	78.24	-0.331	Chloroplast
XM_048480935.1	*Zijuj09G0023200*	1005	334	37.63	5.85	24.49	82.31	-0.234	Chloroplast
XM_048480921.1	*Zijuj09G0023300*	2946	981	110.54	5.77	33.30	80.93	-0.229	Chloroplast
XM_016036388.3	*Zijuj09G0023400*	1527	508	57.17	5.37	24.52	78.48	-0.311	Chloroplast
XM_016036433.3	*Zijuj09G0023500*	3039	1012	114.36	5.88	35.73	83.43	-0.185	Plasma
XM_048481612.1	*Zijuj09G0149600*	1605	534	60.70	6.38	33.89	86.18	-0.290	Vacuolar
XM_048474644.1	*Zijuj05G0053300*	1335	444	50.41	6.40	35.93	91.78	-0.283	Chloroplast
XM_016026995.3	*Zijuj05G0069700*	3849	1282	144.07	8.98	37.85	86.31	-0.264	Chloroplast
XM_048472974.1	*Zijuj05G0089900*	1407	468	53.73	8.84	20.71	75.24	-0.341	Cytoplasm
XM_048474532.1	*Zijuj05G0090100*	1479	492	56.48	5.90	29.64	77.72	-0.316	
XM_048474198.1	*Zijuj05G0113800*	1677	558	63.48	6.62	32.88	80.36	-0.365	Chloroplast
XM_048470813.1	*Zijuj03G0129500*	1209	402	45.64	8.88	30.93	80.80	-0.331	Mitochondria
XM_048468455.1	*Zijuj02G0020900*	1542	513	58.39	6.44	32.18	78.28	-0.284	Chloroplast
XM_048468474.1	*Zijuj02G0041100*	1722	573	65.66	5.05	27.28	73.73	-0.520	Cytoplasm
XM_048468467.1	*Zijuj02G0041600*	1596	531	60.65	5.39	22.80	77.89	-0.475	Cytoskeleton
XM_048468469.1	*ZsBgl40*	1497	498	57.26	5.33	22.36	73.47	-0.564	Cytoplasm

XM_048468471.1	*Zijuj02G0041900*	1497	498	57.32	5.53	21.06	74.24	-0.532	Cytoskeleton
XM_016020484.3	*Zijuj02G0201100*	1599	532	60.96	9.12	29.64	76.86	-0.398	Chloroplast
XM_048468992.1	*Zijuj02G0201200*	3513	1170	132.63	6.62	30.93	77.37	-0.341	Plasma
XM_016020485.3	*ZsBgl03*	1485	494	56.18	8.64	28.23	72.65	-0.449	Chloroplast
XM_016024058.3	*Zijuj01G0002300*	1620	539	60.76	7.60	33.00	78.85	-0.327	Chloroplast
XM_048467470.1	*Zijuj01G0027100*	2247	748	84.32	8.78	26.99	78.69	-0.233	Vacuolar
XM_048471035.1	*ZsBgl24*	1395	464	52.90	9.12	24.91	76.75	-0.260	Chloroplast
XM_048469917.1	*Zijuj01G0027900*	1497	498	56.22	8.98	29.03	78.31	-0.252	Chloroplast
XM_048468178.1	*Zijuj01G0028000*	2242	813	92.73	9.16	24.83	78.87	-0.226	Plasma
XM_025073468.2	*Zijuj01G0028100*	1488	495	56.10	6.97	25.00	78.61	-0.278	Chloroplast
XM_048469777.1	*Zijuj01G0129400*	1368	455	52.22	6.37	32.33	81.45	-0.311	Cytoplasm
XM_016029321.3	*Zijuj01G0129700*	1467	488	55.45	8.20	28.12	78.40	-0.287	Cytoplasm
XM_048469759.1	*Zijuj01G0218900*	1518	505	58.15	9.04	24.46	82.30	-0.345	Cytoplasm

Table S4 Mass data for the 15 saponins detected in the Ziziphi Spinosae Semen extract by UPLC-MS/MS

NO.	t_R/min	Chemicals	Molecular Formula	Selected ion	Theoretical	Experimental	Error (ppm)	Fragment ions (m/z)
1	1.60	Protojujuboside B	$C_{58}H_{96}O_{27}$	[M+HCOO]-	1269.6110	1269.6124	1.103	1052.9314，908.6953，851.1861，787.4107，720.9116，625.3610，523.7327，479.3085
2	1.74	Jujuboside G	$C_{52}H_{86}O_{22}$	[M+HCOO]-	1107.5581	1107.5592	0.994	929.4955，911.4881，787.4267，776.1662，749.4559，654.6995，625.3499
3	2.81	Jujuboside E	$C_{64}H_{106}O_{31}$	[M+H]+	1371.6790	1371.6926	9.920	1058.6342，603.2946，311.0053，227.8897
4	4.05	Jujuboside A	$C_{58}H_{94}O_{26}$	[M-H]-	1205.5949	1205.5852	-8.050	1097.3269，1073.5752，927.4910，911.4881，893.4856，749.4574，603.3928，463.4588，
	4.19			[M+HCOO]-	1251.6004	1251.6013	0.719	1235.6085，1089.5552，1055.5604，1073.5364，927.4910，893.4856，911.5211，795.6090，749.4534，603.3928
5	4.33	Jujuboside C	$C_{59}H_{96}O_{27}$	[M-H]-	1235.6055	1235.6071	1.296	1097.3269，1073.5852，927.4910，911.4881，893.4856，749.4574，603.3928，463.4588，
	4.10			[M+HCOO]-	1281.6110	1281.6119	0.703	466.4201，271.6797，227.8861
6	4.08	Jujuboside D	$C_{58}H_{94}O_{26}$	[M-H]-	1205.5949	1205.5852	-8.050	1073.5421，911.5211，879.4148，749.4549，603.3928
	4.91			[M+HCOO]-	1251.6004	1251.6013	0.719	1235.6123，1073.5391，434.9091，357.6076，329.7279
7	4.10	Jujuboside IV	$C_{58}H_{94}O_{26}$	[M-H]-	1205.5949	1205.5852	-8.050	1057.6070，1176.4395，1073.5395，1055.5634，927.4910，911.4923，846.8998，819.4412，749.4550，731.7894，611.4460，603.3928
	5.55			[M+HCOO]-	1251.6004	1251.6013	0.719	1174.6004，1073.5391，272.0080
8	6.58	Jujuboside II	$C_{52}H_{84}O_{21}$	[M-H]-	1043.5421	1043.5334	-8.341	911.4984，749.4479，603.3948
	6.59			[M+HCOO]-	1089.5476	1089.5493	1.561	1043.5443，911.5008，740.4478，603.3910，567.3691，471.3478，317.2489
9	6.64	Jujuboside B	$C_{52}H_{84}O_{21}$	[M-H]-	1043.5421	1043.5334	-8.341	911.5051，893.4988，749.4480，603.3936，488.6169，

								406.1642
	6.97			[M+HCOO]$^-$	1089.5476	1089.5493	1.561	911.4937，749.4554，603.3898，503.7469，353.0318
10	6.68	Jujuboside I	$C_{53}H_{86}O_{22}$	[M+HCOO]$^-$	1119.5581	1119.5586	0.447	911.5040，749.4464，603.3979，550.4784
11	6.73	Unknown	$C_{53}H_{86}O_{22}$	[M+HCOO]$^-$	1119.5581	1119.5586	0.447	1073.5391，911.4881，749.4563，714.9927
12	7.84	Zizyphus saponin II	$C_{47}H_{76}O_{17}$	[M+HCOO]$^-$	957.5053	957.5068	1.567	872.8751，513.2857，397.8773，378.5062，367.3773
13	6.58	Jujuboside B1	$C_{52}H_{84}O_{21}$	[M-H]$^-$	1043.5421	1043.5334	-8.341	911.4851，749.4530，603.3928，232.9637
	7.07			[M+HCOO]$^-$	1089.5476	1089.5493	1.561	911.4881，749.4588，603.3928，503.7429，353.0314
14	6.64	Jujuboside III	$C_{52}H_{84}O_{21}$	[M-H]$^-$	1043.5421	1043.5334	-8.341	911.4896，893.4856，749.4527，603.3928，488.6161，406.1622
	8.22			[M+HCOO]$^-$	1089.5476	1089.5493	1.561	1043.5448，911.5005，893.4946，765.4118，749.4476，713.1871，656.4776，603.3931
15	12.85	Acetyljujuboside B	$C_{54}H_{86}O_{22}$	[M+HCOO]$^-$	1131.5581	1131.5602	1.857	1138.3371，911.5211，893.4856，749.4519，603.3928

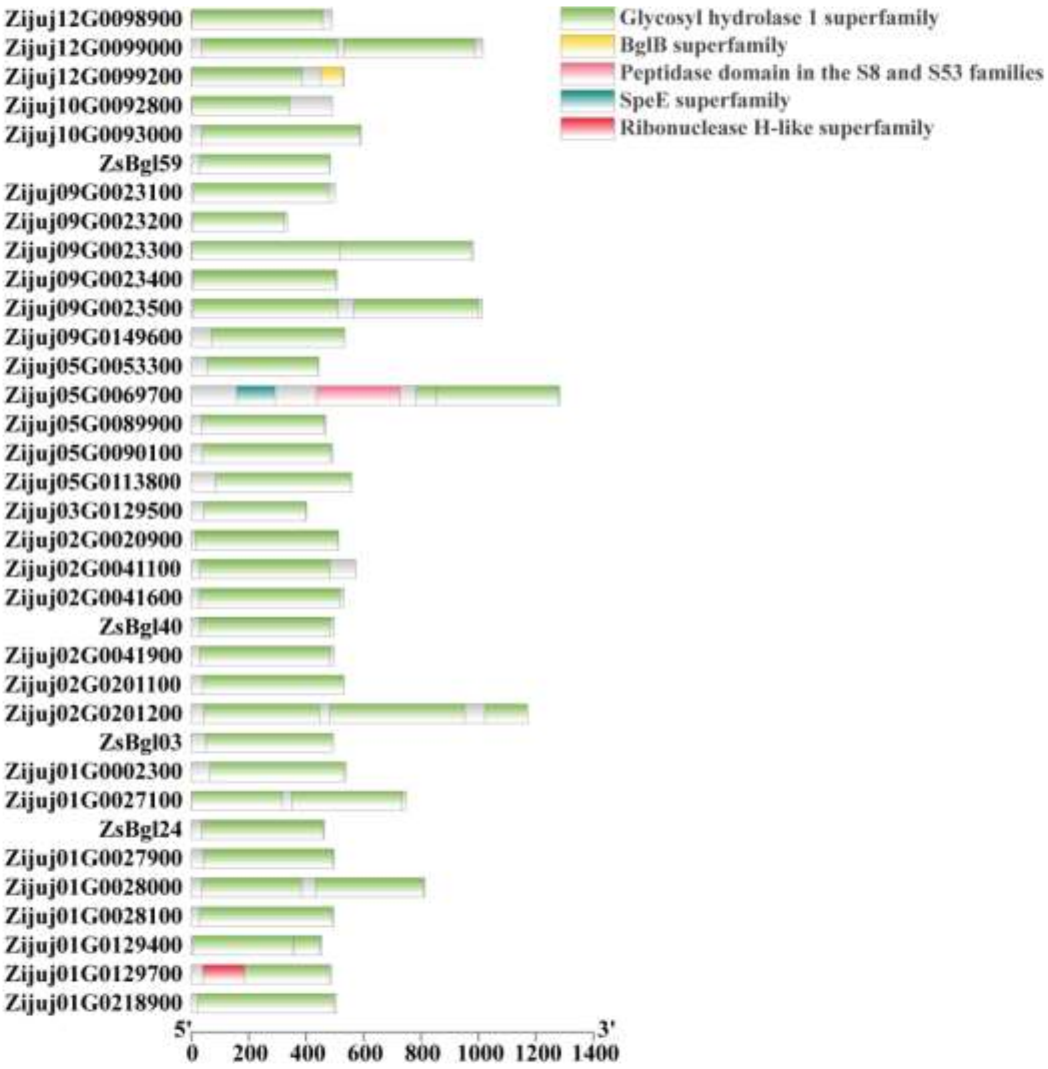

Figure S1. The major domains of the putative ZsBgl proteins

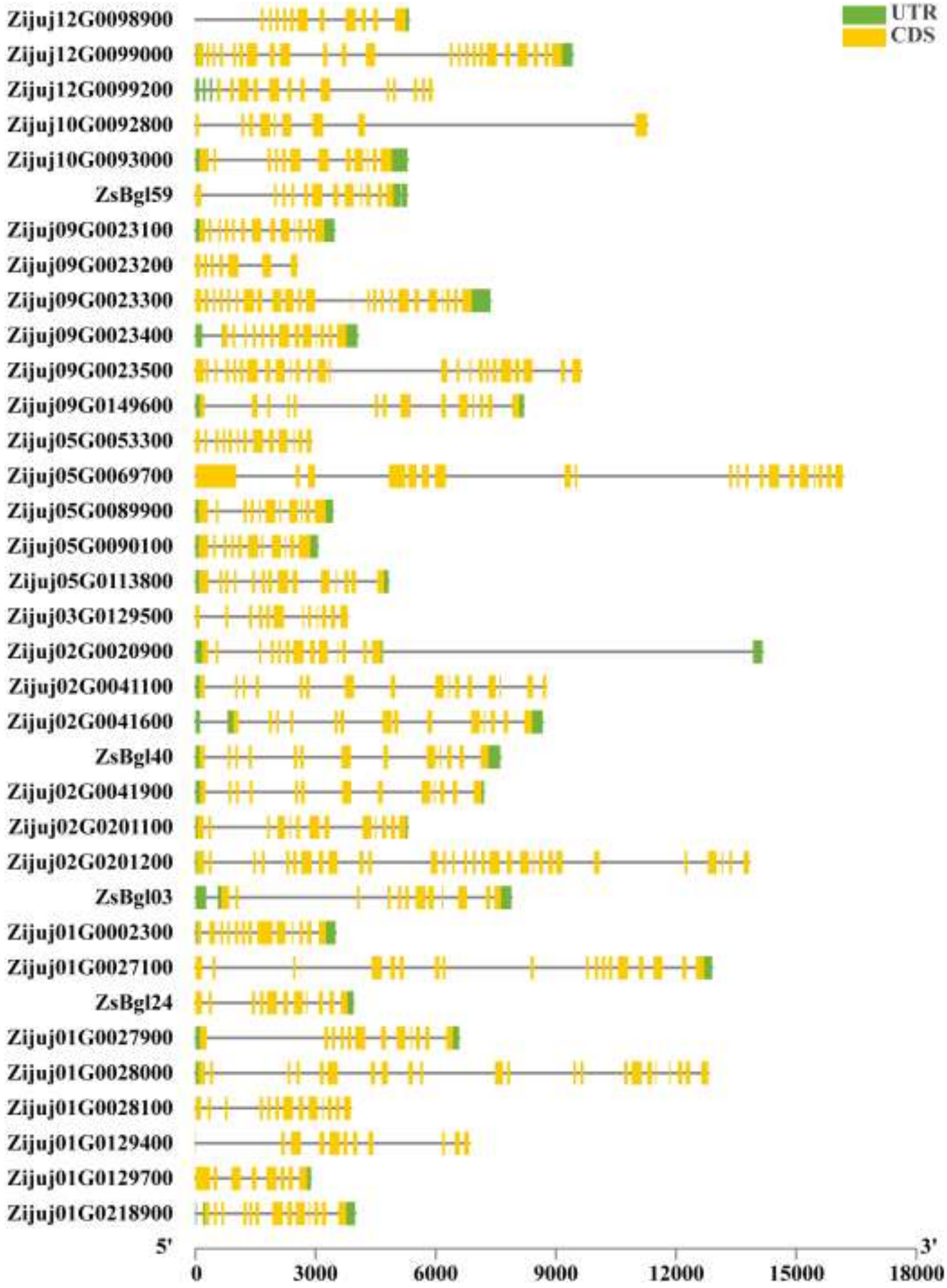

Figure S2. The exon/intron structures of the *ZsBgl* genes

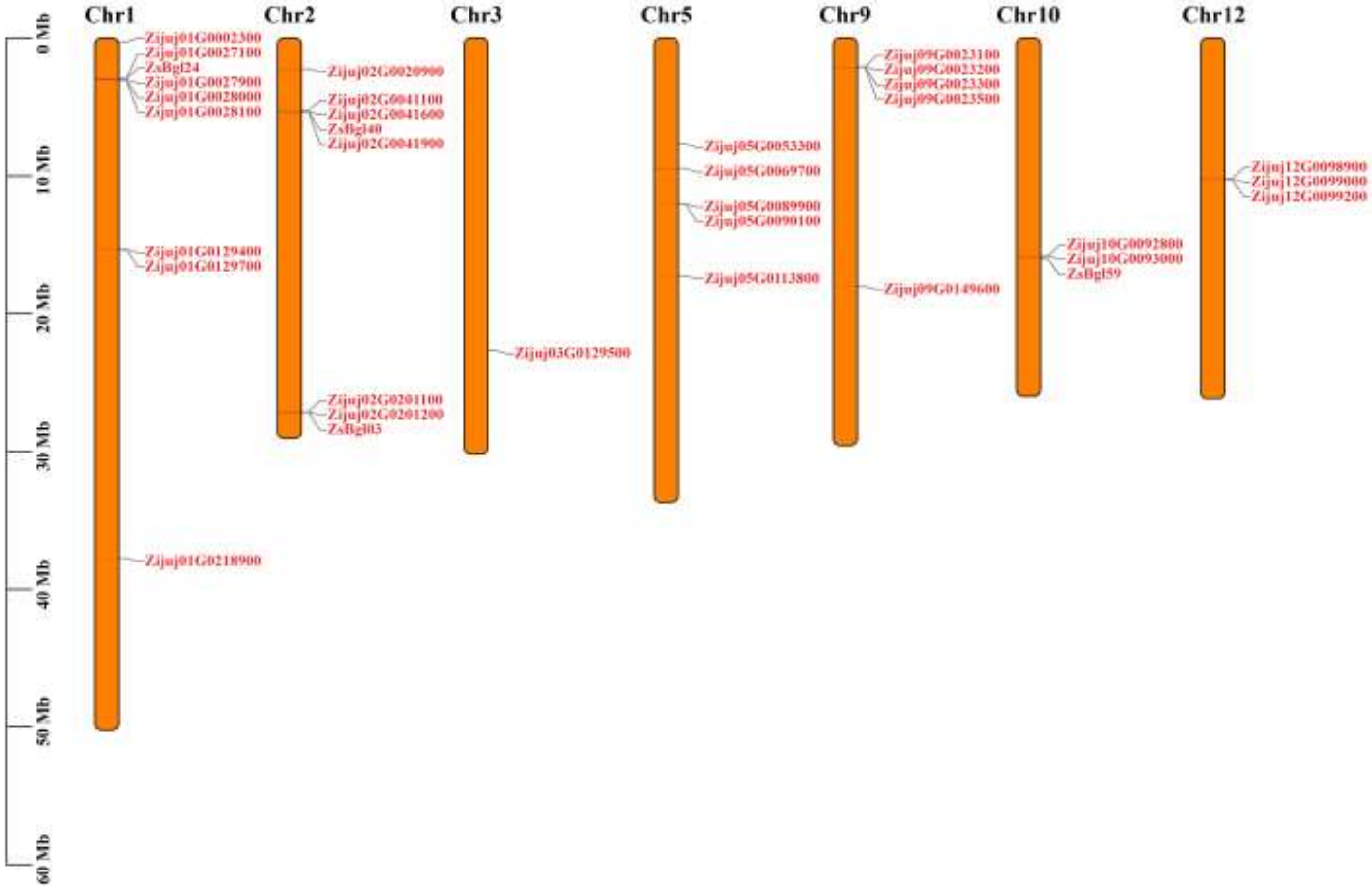

Figure S3. The locations of the *ZsBgl* genes on the chromosomes

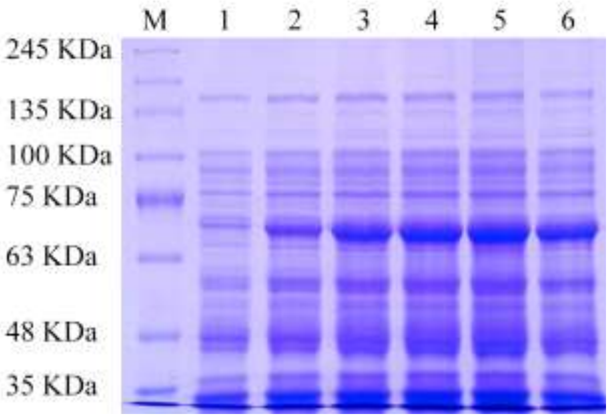

Figure S4. Detection the heterologous expression of ZsBgl03 through SDS-PAGE. The Coomassie Brilliant Blue stained gel shows the expression changes of ZsBgl03 upon the IPTG induction for a short time. M: Protein molecular weight marker; 1: Proteins produced from *E. coli* transformed by the empty vector pET32a; 2: Proteins produced from the recombined *E. coli* carried *ZsBgl03*; 3-6: Proteins produced from the recombined *E. coli* carried *ZsBgl03* by IPTG induction for 1 h, 2 h, 3 h, and 4 h, respectively.

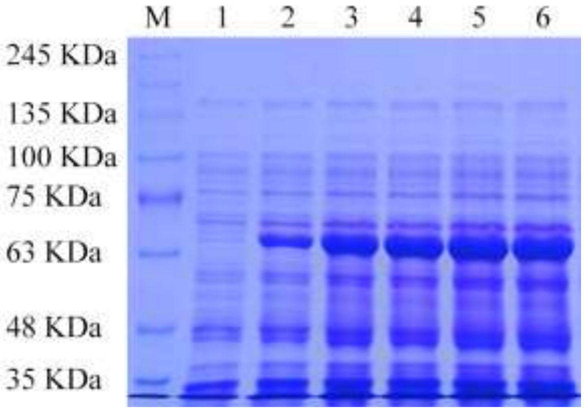

Figure S5. Detection the heterologous expression of ZsBgl40 through SDS-PAGE. The Coomassie Brilliant Blue stained gel shows the expression changes of ZsBgl40 upon the IPTG induction for a short time. M: Protein molecular weight marker; 1: Proteins produced from *E. coli* transformed by the empty vector pET32a; 2: Proteins produced from the recombined *E. coli* carried *ZsBgl40*; 3-6: Proteins produced from the recombined *E. coli* carried *ZsBgl40* by IPTG induction for 1 h, 2 h, 3 h, and 4 h, respectively.

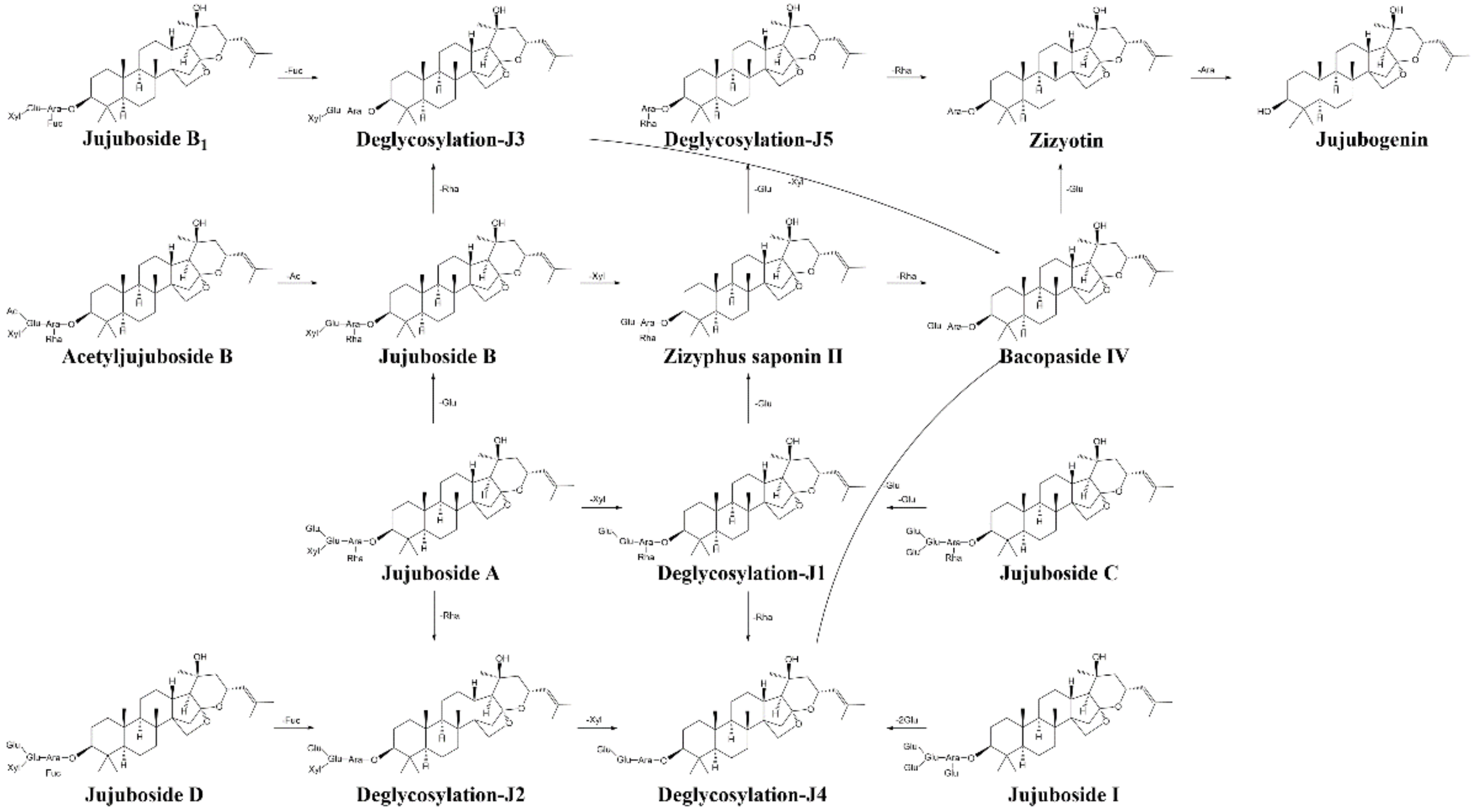

Figure S6. The biotransformation of jujubosides

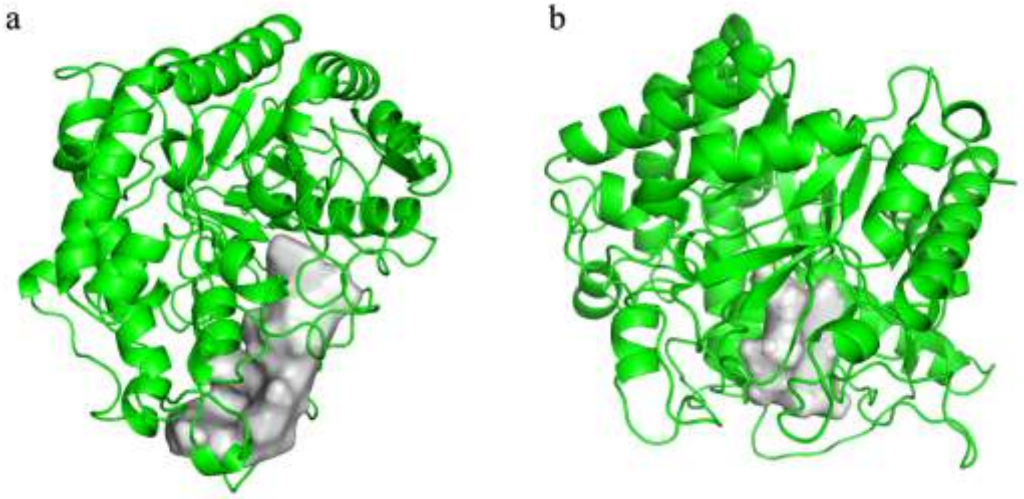

Figure S7. The catalytic pocket and position in the tertiary structure of ZsBgl03 and ZsBgl40 predicted by the online tool POASA 1.1. (**a**) ZsBgl03. (**b**) ZsBgl40. Green represents the predicted protein, and gray represents the active pocket. This pocket is a large highly conserved catalytic pocket that excludes the possibility of false pockets based on volume selection and visual analysis to predict the binding site. The binding site of the small molecule ligand is located in the pocket.
